# Endoscopic Transaxillary Breast Augmentation – a Case Series of 1300 Patients

**DOI:** 10.1093/asjof/ojae007.035

**Published:** 2024-04-12

**Authors:** Yaeesh Sardiwalla, Shim Ching

**Affiliations:** McMaster University, Hamilton, Canada; Asia Pacific Plastic Surgery, Honolulu

## Abstract

**Level of Evidence:** **V – case series**

**Goals/Purpose:**

Breast augmentation is currently one of the most common aesthetic surgical procedures performed in the United States. Transaxillary breast augmentation offers an advantageous hidden scar compared with other options for implant placement. While first described as a blunt and blind approach, endoscopic technique further refined this procedure to offer surgeons complete control and visualization of dissection for precise implant placement. Despite this, breast augmentation is done far more commonly as was initially described over 60 years ago with incisions on the breast that can result in visible scarring.

The purpose of this study was to report surgical outcomes on the largest published series to date in a patient cohort of 1389 endoscopic transaxillary breast augmentations. We describe the refinement of this technique and statistical analysis of patient outcomes.

**Methods/Technique:**

This study included all patients with breast hypoplasia who chose endoscopic transaxillary breast augmentation from 2006 to 2022 by a single surgeon (S.C.). All patients were followed for a minimum of 3 months post procedure. All implants used were smooth, round, silicone gel implants with volumes ranging from 180 to 800cc. Implants were placed in the subfascial or submuscular plane.

An incision was marked in the most prominent axillary crease. If there were no creases, an incision was marked at the highest point of the armpit along Langer’s lines. A 30 degree endoscope (Karl Storz, USA) was placed in an endoscopic breast retractor (Karl Storz, USA) and dissection proceeded either in the submuscular plane between the pectoralis major and minor, or in the subfascial plane with an angled suction cautery (Black and Black Surgical, Texas). In the case of subfascial implant placement, the pocket was dissected according to the preoperative markings. With submuscular placement, pocket dissection also followed markings, but the pectoralis muscle was divided with cautery from the level of the areola along the sternal origins of the muscle to completely divide the muscle inferiorly and laterally.

The inframammary fold was lowered as necessary by cautery dissection above the level of the pectoralis fascia inferiorly. Glandular scoring was performed with cautery when needed as well. After dissection was completed, saline breast implant sizers were inserted and filled with air to estimate implant size and confirm pocket dissection in the upright position. Adjustments were made to the implant pocket with endoscopic cautery dissection as was necessary.

**Results/Complications:**

A total of 1389 patients were included in our data analysis for surgeries performed between March 2006 and December 2021. Overall complication rate in our cohort was 6.69%. Malposition of implants was the most common complication at 3.64%. Contracture rate was 1.74%. There was a significant increase noted in hematoma rate in subfascial placement (3.51% increase, p-value<0.05) compared to submuscular and a decrease in implant malposition in the subfascial group (6.58% decrease, p-value<0.05) compared to the submuscular group. These complications were largely managed using endoscopic techniques. There were no significant differences noted between subfascial, subglandular and submuscular pocket placement in asymmetry, contracture and hypertrophy.

**Conclusion:**

We describe a safe and effective approach to endoscopic transaxillary breast augmentation that has demonstrated long term results compared to the direct vision inframammary technique. The advantages of a hidden scar should be a consideration for surgeons to adopt this technique to improve patient outcomes.

